# Need for and design of a trial to test efficacy of weight loss interventions for cancer prevention: an international consensus using expert nominal group and Delphi methods

**DOI:** 10.1038/s41416-026-03356-7

**Published:** 2026-03-02

**Authors:** Matthew Harris, David P. French, Ken Clare, Michelle Harvie, Duncan T. Wilson, Julia Brown, David Jayne, Andrew G. Renehan

**Affiliations:** 1 Division of Cancer Sciences, School of Medical Sciences, Faculty of Biology, Medicine and Health, University of Manchester, Manchester, UK; 2 Division of Psychology and Mental Health, Faculty of Biology, Medicine and Health, University of Manchester, Manchester, UK; 3 Obesity Voices, Obesity Institute, Leeds Beckett University, Leeds, UK; 4 Nightingale/Prevent Breast Cancer Centre, Wythenshawe Hospital, Manchester University NHS Foundation Trust, Manchester, UK; 5 Leeds CRUK Clinical Trials Unit, Leeds Institute of Clinical Trials Research, University of Leeds, Leeds, UK; 6 Leeds Institute for Medical Research, University of Leeds, Leeds, West Yorkshire UK; 7 Manchester Cancer Research Centre, NIHR Manchester Biomedical Research Centre, Manchester, United Kingdom

**Keywords:** Cancer prevention, Cancer epidemiology, Clinical trials, Metabolic disorders

## Abstract

Obesity is associated with increased risk of at least 13 cancer types. Evidence from bariatric surgery cohorts and some behavioural intervention trials supports the notion that weight loss can prevent obesity-related cancers. The introduction of glucagon-like peptide (GLP)-1 agonist drugs has rapidly revolutionised pharmacotherapy options. A cancer prevention clinical trial would be complex, lengthy, and costly; therefore, we undertook an international expert consensus to assess the need for and design of a weight-loss intervention cancer prevention trial. We used a combination of two nominal group meetings, sandwiching 3 Delphi rounds. A panel of 54 international, multi-disciplinary researchers was established, informed by patient groups. Feedback was incorporated iteratively, and borderline statements, those that did not reach consensus, were addressed in a final meeting. Through the Delphi rounds, retention rates were high (98%, 85%, 88%). Consensus was achieved on 25 statements. Including: (i) there is a need for clinical trial evidence to inform obesity-related cancer prevention strategies; (ii) a trial should reflect high-risk populations; (iii) trials should prioritise GLP-1 agonists; and (iv) future research should explore mechanistic pathways and relevant cancer precursors. This consensus underscores the need for trial evidence to inform strategies for obesity-related cancer prevention.

## Background

Excess body weight (EBW), commonly approximately as body mass index ≥25 kg/m^2^ (termed overweight and obesity) is associated with increased risk of at least 13 adult cancer types [1]. These obesity-related cancers are: oesophageal adenocarcinoma, gastric cardia, colorectal, liver, gallbladder, pancreas, post-menopausal breast, corpus uteri, ovarian, renal-cell, thyroid cancers, meningioma and multiple myeloma [1]. In many western populations, EBW is now the second commonest cause of cancer after smoking attributing to ~5% of cancers in men and 9% in women [2]. In parallel, over the past 30 years, the prevalence of EBW doubled in women and tripled in men globally [3]. Thus, there is a need to consider evidence to inform obesity-related cancer prevention strategies.

Evidence from observational studies of bariatric surgery [4, 5] (where typical sustained 10 year weight loss is ~22% [6]), and to a limited extend, from randomised trials of behavioural interventions [7, 8] (where long-term weight loss is generally 3–5%), support the notion that weight loss interventions prevent obesity-related cancers. In the past five years, the introduction of glucagon-like peptide (GLP)-1 agonist family of drugs as obesity management interventions (where 2–4 year weight loss is approximately 15% with Semaglutide, a GLP-1 agonist [9]; ~25% with Tripeptide, a GLP-1 dual agonist [10]; and ~24% with Retatrutide, a triple agonist [11]) has rapidly and dramatically revolutionised a pharmacotherapy option as a weight losing intervention. To date, there are no GLP-1 agonist intervention trials where cancer prevention is the primary interest. Thus, the effect of pharmacological weight loss on cancer risk is poorly understood and not yet considered in cancer prevention clinical practice guidelines and policy.

Here, we address the question whether a clinical trial testing the efficacy weight loss intervention on cancer incidence would be informative as a prevention strategy. Such a trial is likely to be challenging for multiple reasons: long duration; rarity of incident cancers and therefore large sample sizes; complexity of non-surgical weight loss intervention; and high financial cost. Any trial in this scenario must undergo a thorough assessment of need and feasibility to avoid damaging failure. As this is a rapidly changing field, we chose to undertook an international expert consensus to assess the need for and design of a weight loss intervention cancer prevention trial.

## Methods

We used a combination of 3 Delphi rounds [12, 13], sandwiched by two expert nominal group meetings [14], to achieve consensus. We reported in accordance with the ACCORD guidelines [15]. We did not register a study protocol, as consensus studies are often not registered prior to commencement.

### Selection of the steering committee

**Fig. 1:**
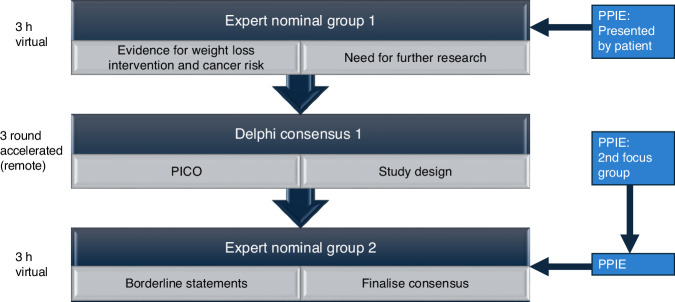
The overall study design process combines Expert nominal group and Delphi methods. PICO Population Intervention Comparison Outcome, PPIE Public and patient Involvement and Engagement.

We formed a study steering committee (SC), with represented diverse disciplines including oncology (MJH, AGR),  clinical trials (JB), behavioural science (DF), dietetics and weight management (MH), statistics (DW), surgery (DJ) and patients (KC). Given the complexity of the area and need for broad multi-disciplinary input of participants, a decision to have 3 stages was taken, with more inductive elements at the start (rather than systematic review), allowing a focussed Delphi process to consider agreement with these elements (Fig. 1). Throughout the process, public and participant involvement and engagement (PPIE) groups were held to inform statements and a PPIE representative was present through the process (KC) at the expert nominal group meetings.

### Stage 1 (preparatory): Expert nominal group 1

A three-hour virtual meeting was held in March 2024, which included multi-disciplinary researchers invited by selection of the steering committee (Table 1). Researchers were invited based on expertise in obesity and cancer research and as representatives of a multi-disciplinary demographic with the sample size of 19 defined by a balance of nominal group best practices [14] and the requirement for a diverse panel. This group aimed to define the need for a consensus on weight-loss interventions and cancer prevention and the content of a Delphi consensus. Group discussions were held to elicit themes for Delphi consensus statements. PPIE group findings were presented to the group prior to commencement and the PPIE consensus advisor was present, KC, chair of the European Coalition for People living with Obesity (ECPO, https://eurobesity.org/).

**Table 1:** Self-identified demographics of the expert nominal groups 1 and 2.

Category	Demographic	Nominal group 1 (*n* = 19)	Nominal group 2 (*n* = 30)
	Event dates	14/03/2024	21/10/2024
Country of affiliation	United Kingdom	16	20
	Ireland	1	1
	France	1	0
	Sweden	0	1
	Italy	0	1
	Germany	0	2
	Switzerland	1	1
	Denmark	0	1
	USA	0	2
	Australia	0	1
Research area	General surgeon	3	2
	Bariatric surgeon	1	1
	Endocrinologist/obesity physician (clinical)	2	6
	Evidence synthesis organisation	2	2
	Cancer clinical trials	1	1
	Statistician	2	1
	Cancer epidemiologist	2	3
	Dietician	1	1
	Physiotherapist	0	1
	Public health (cancer)	2	0
	Gynaecologist	0	1
	Basic science (cancer)	0	5
	Cancer molecular epidemiology	0	2
	Behavioural science	3	4
Gender	Male	11	14
	Female	7	16

### Stage 2: Delphi consensus

A 3-round remote Delphi consensus was held with statements informed from Nominal Group 1 and PPIE. Participants were invited based on expertise in cancer and obesity research across the world. Broad multi-disciplinary representation was a priority, and participants were invited via a combination of personal invitation and through networks, including the European Association for the Study of Obesity (EASO), and the International Federation for the Surgery of Obesity and metabolic disorders (IFSO), with sample sizes finalised after ensuring good representation from all major disciplines and leaders in the field. Invited participants received a summary of nominal group 1 discussions and a summary of literature to provide context to the consensus, obtained through a thorough literature review prior to commencement of the consensus.

There were no financial incentives to participate in the Delphi. The Delphi was solely in English language. Each round was held on Google Forms™ software (https://workspace.google.com/intl/en_uk/products/forms/) with all responses anonymised with data stored on secure University servers and only accessible to core members of the steering committee (MH, AGR). Participants were presented with the option to either ‘strongly agree’, ‘agree’, ‘neither agree nor disagree’, ‘disagree’ or ‘strongly disagree’. Due to the broad expertise of participants, complexity of the statements and plan for further nominal group discussion of borderline statements, consensus was defined as 70% of participants selecting agree and strongly agree or disagree and strongly disagree or neither agree not disagree. Borderline consensus was defined as 65–75%. The response ‘nether agree nor disagree’ was considered independent and able to reach consensus itself.

Alongside each new round, the participant received the results of the previous round, along with their response in that round. Free text feedback was encouraged in round 1 and 2 and this was used to inform the addition or amendment of statements and clarification to wording of existing statements. This approach was taken to allow for the elicitation of important topics from a broad area of research and expertise. The start dates of each round were June 2024, July 2024, August 2024 (Table 2).

**Table 2:** Self-identified demographics of the Delphi panelists, per voting round.

Category	Demographic	Delphi round 1 (*n* = 54)	Delphi round 2 (*n* = 46)	Delphi round 3 (*n* = 48)
	Event dates	19/06/2024 to 04/07/2024	15/07/2024 to 30/07/2024	06/08/2024 to 09/09/2024
Country of affiliation	United Kingdom	28	26	24
	Ireland	2	2	1
	France	1	0	0
	Sweden	1	1	1
	Italy	1	1	1
	Germany	3	3	3
	Switzerland	10	7	8
	Denmark	3	2	2
	USA	3	2	2
	Australia	2	2	2
Research area	General surgeon	2	2	2
	Bariatric surgeon	7	4	5
	Endocrinologist/obesity physician (clinical)	11	10	10
	Evidence synthesis organisation	3	2	1
	Cancer clinical trials	3	3	3
	Statistician	2	2	2
	Cancer epidemiologist	4	3	4
	Dietician	2	2	2
	Physiotherapist	1	1	2
	Public health (cancer)	3	3	3
	Gynaecologist	2	2	2
	Basic science (cancer)	5	3	4
	Cancer molecular epidemiology	4	4	4
	Behavioural science	3	3	3
Gender	Male	30	24	26
	Female	24	22	22

The Delphi was structured into six domains – four of which were based around the PICO (Population, Intervention, Comparison, and Outcome) [16], and were: the need for a clinical trial; population considerations; intervention considerations; comparison considerations; outcome considerations; and future research priority areas.

### Stage 3: Expert nominal group 2 consensus meeting

Following completion of the Delphi, all participants were invited back to a second group meeting and received the results of the Delphi consensus. At this meeting (October 2024), there was an opportunity to discuss the results in general, each borderline statement specifically and areas that did not reach consensus. Three members of the SC (MJH, AGR, MH, DF) were included in the consensus meeting. Attendees were then able to undertake live voting for each borderline statement following discussion. Group discussion on areas that did not reach consensus was held to allow for the generation of new statements, on which participants were able to vote.

## Results

### Stage 1: Nominal group 1

This first virtual meeting was attended by 19 international and multi-disciplinary researchers, including the study lead (MJH) and 2 facilitators (DF, JB) with a 100% response to invitations sent. Participants in the nominal group 1 meeting are listed in Supplementary Table S1, including area of discipline and University affiliation. During this meeting, there was agreement on: (i) the current evidence for the causal relationship between weight-loss intervention to prevent cancer is not strong enough; and (ii) there is a need to explore further research in this area and that a clinical trial is necessary to build on the current evidence for weight-loss intervention to prevent cancer. Qualitative themes were also elicited to inform the Delphi consensus statements.

### Stage 2: Delphi consensus

60 international researchers were invited, of whom 54 responded. 52 (96%) participants took part in the first round, with 46 (85%) and 48 (89%) in round 2 and round 3, respectively (Tables 1 and 2). All returned questionnaires were returned with 100% completion, and therefore, there was no variable missing data between participants. Participants were from 12 countries, with the majority from the UK and EU. There was a good representation of expertise with a blend of clinicians, pre-clinical researchers, behavioural scientists, endocrinologists,dietitians, epidemiologists, statisticians and clinical trialists. The full list of participants is available in Supplementary Table S2, and the breakdown of geographical area and expertise is available in Supplementary Tables 1, 2.

**Fig. 2:**
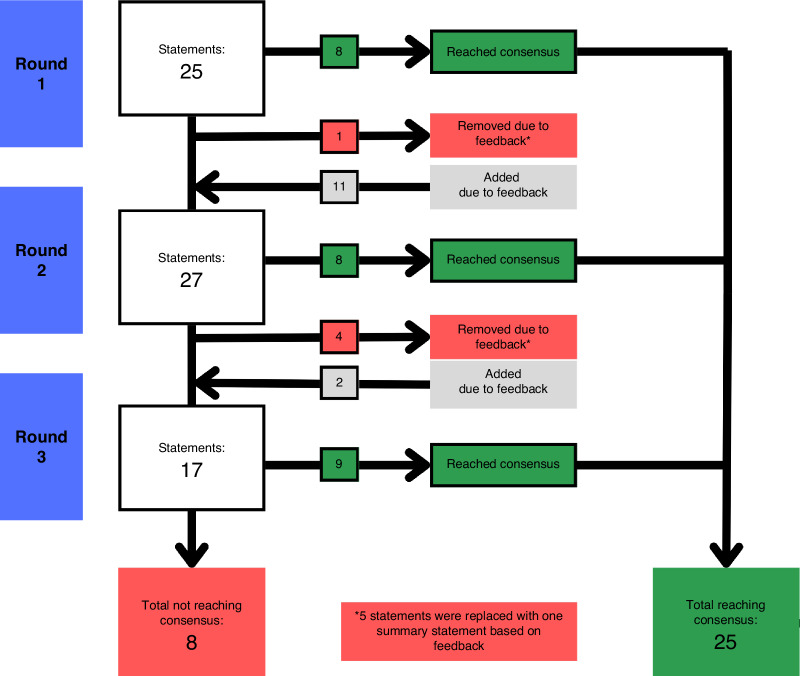
A flow diagram to show the development of consensus items.

38 individual statements were asked, with 25 reaching consensus, 5 removed and 8 not reaching consensus. The 5 that were removed were all regarding the selection of control groups for specific trial scenarios. Following feedback, they were replaced with one statement that reached 100% consensus in its first round. Of the 8 that did not reach consensus, all were explored during stage 3. 7 statements were defined as borderline and were explored during stage 3 of the consensus. The flow of statements through each round is illustrated in Fig. 2. Full results from the Delphi are available in Supplementary Table S3.

Consensus was reached that a clinical trial is necessary to advance the current evidence for the causal effect for weight-loss intervention and cancer risk reduction and that such a trial should have a follow-up of 10 or more years. There was agreement that a population in this trial should be broadly representative of the UK/EU and should consider a generic high baseline cancer risk, such as BMI > 40, but not specific risks such as genetic predisposition. There was a preference for assessing GLP-1 or dual receptor agonists as the intervention and that obesity-related cancer and all cancer should be considered as primary endpoints, either in combination or alone. There was strong agreement on the consideration of cancer precursors as surrogate endpoints for cancer incidence. Further research priority areas were identified including understanding the biological causal pathways between weight-loss intervention and cancer development, the identification of cancer precursors and the impact of weight-loss intervention on molecular, hormonal, metabolic and inflammatory pathways in relation to cancer development.

### Consensus meeting

31 participants attended the second nominal group meeting (list available in Supplementary Table 4). Of the 7 Delphi statements that were borderline, 4 underwent group discussion and live voting, leading to inclusion of 3 and exclusion of 1 (Table 3). Two small group discussions were held to explore 8 statements that did not reach consensus and the remaining 3 borderline statements that did not reach the pre-defined consensus threshold during the Delphi, including: ‘the need for evidence to inform policy’ and ‘the delivery of GLP-1/dual receptor agonists in a clinical trial’. These discussions generated two new statements that underwent live anonymous voting as a group. Both new statements achieved strong consensus and are included in the recommendations.

**Table 3:**
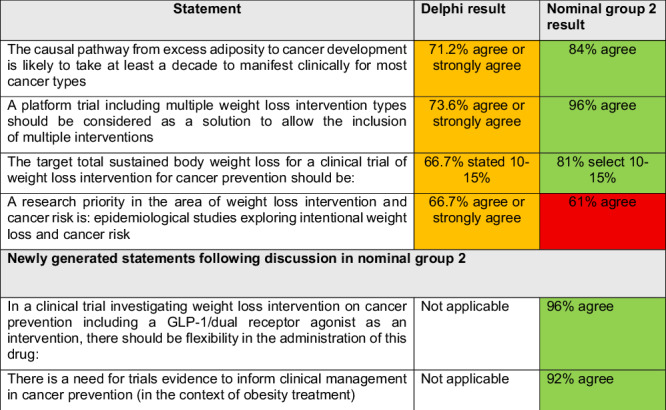
Borderline statements after 3 rounds of Delphi and new statements voted on in nominal group meeting 2.

Green shade: consensus IN; Ample shade: borderline; Red shade: consensus OUT.

During this stage, group discussion generated consensus was achieved that clinical trial evidence is needed to inform clinical management policy in the context of obesity and cancer prevention. There was also consensus agreement that there should be flexibility in the administration of the drug intervention if GLP-1 or dual agonists were to be used in this context.

### Final consensus recommendations

37 statements were explored in the Delphi process, with 25 reaching consensus and being included as recommendations. 2 new statements generated from nominal group meeting 2 were added, to create a final list of 27 consensus statements shown in Table 4.

**Table 4:**
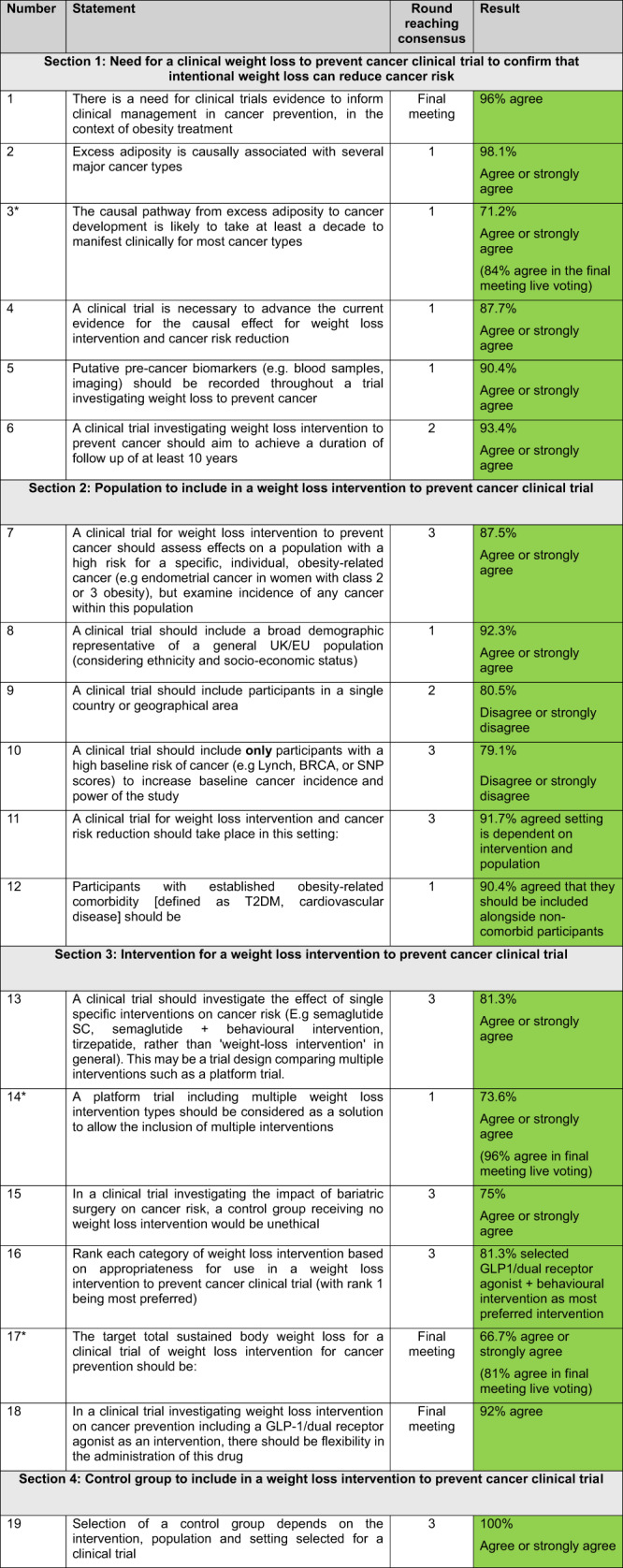

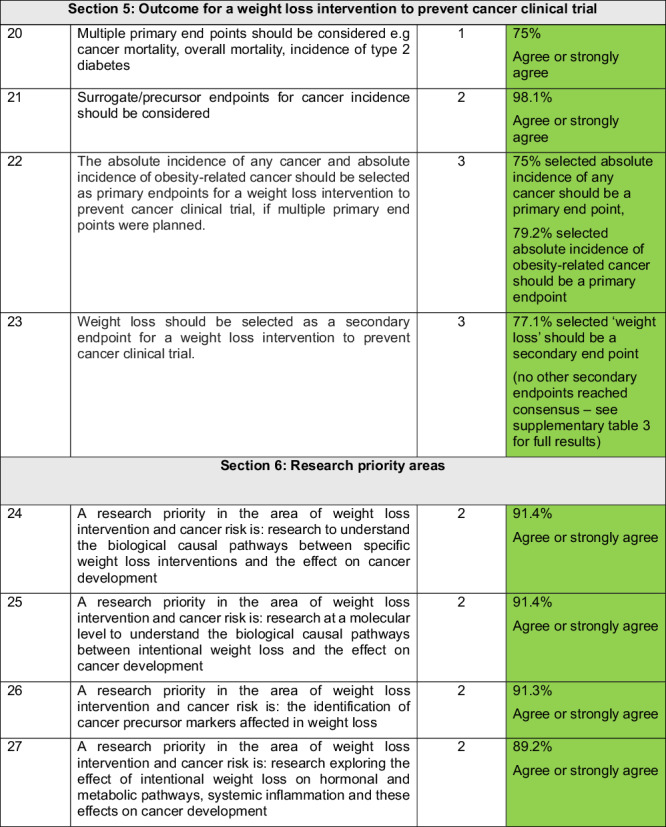
Consensus on the need for a clinical trial investigating weight loss intervention to prevent cancer.

^*^Borderline statement that was voted on in nominal group 2.

Green shade: consensus IN.

## Discussion

### Main findings

This consensus process has highlighted the widespread agreement on the need for a trial to investigate weight-loss interventions and the potential cancer prevention effects, to guide future clinical management guidelines. It has been highlighted that a trial needs to have long-term follow-up in the order of 10 years or more, and that the absolute incidence of cancer or obesity-related cancer should be the primary outcome. GLP-1/dual receptor agonists have been identified as a promising intervention of interest and priority in this context. An ideal clinical trial should include participants that are broadly representative of a UK, EU and US population, to maximise the generalisability of the results. A long-term trial with large sample sizes required to identify a difference in absolute cancer incidence will be financially costly. Therefore, the need for the identification of cancer precursors or surrogate endpoints and the consideration of their use in this context may be key to undertaking a trial and mitigating some of these financial challenges. Further research priority areas were identified around understanding the mechanistic aspects of intentional weight-loss on the carcinogenic and the biological causal pathways of specific interventions on cancer development.

### Context of the rest of the literature

GLP-1 receptor agonists have been identified as a potential cancer prevention strategy to be examined in a clinical trial. However, they may provide some logistical and ethical challenges that may influence possible trial designs. Current evidence of the effect of GLP-1 receptor agonist administration has been conflicting. Some studies suggested that there may be an increased risk of thyroid [17] or pancreatic cancer [18]. However, these findings were limited to observational studies including GLP-1 receptor agonists used in the treatment of diabetes and subsequent studies have not replicated these findings [19, 20]. Studies of semaglutide have not shown any significant effect on cancer incidence [21]. The SELECT trial did not show any difference in cancer incidence after a mean follow-up of 3.3 years [22]. Until recently, the focus of research has been of safety, rather than the possible utility of GLP-1 receptor agonists as a cancer prevention strategy. One recent cohort study has shown significant reductions in the risk of several common cancers [23], however current observational evidence is limited by methodological biases [24]. Long-term administration of this category of drugs is not well understood in part due to their novelty and this highlights the need for further research.

In the past, clinical trials have been transformational in identifying medications for the primary prevention of some cancers, for example, post-menopausal breast cancer [25, 26] and colorectal cancer in Lynch syndrome [27]. However, there is no clear signal for obesity-related cancer prevention in current RCT or observational evidence. The LEADER trial, comparing liraglutide to placebo in patients with type 2 diabetes and high cardiovascular risk, with a medium follow-up of almost 4 years did not show a significant difference in neoplasm incidence, strengthening the evidence that GLP-1 drugs do not lead to higher neoplastic rates and alternative drugs that provide higher levels of weight loss, may contribute to a reduced obesity-related cancer risk.

### Strengths and limitations

Strengths included the following. First, this consensus brings together a broad, multi-disciplinary panel of experts in obesity and cancer research. The three-step process allowed for multiple points of refinement and inclusion of a wide range of expertise and ideas. Second, the Delphi consensus was designed to be able to develop with the input of participants and was successful in achieving consensus in a large proportion of statements presented. Third, the final group meeting allowed for exploration of all areas that did not reach consensus and to understand themes and generate supporting recommendations based on exploration. Fourth, public and participant groups were held throughout the consensus process to give insights from those with lived experience and to inform each step of the consensus.

There were limitations. First, the group of researchers was largely UK and European, which may have influenced the results of the consensus, leading to under-representation from certain regions. However, time and funding constraints prevented further expansion despite efforts to recruit more diverse participants. Second, the whole group was not present in nominal group 1, potentially leading to bias in statements presented to the Delphi. Third, due to the number of rounds of nominal group and Delphi meetings, there was attrition at group meeting 2, with 31 of 54 participants attending the live meeting. This could create bias in live-voted statements; however, the final list of consensus recommendations was presented to all Delphi participants for any concerns. Furthermore, we did not explore the practical considerations of funding such a trial, including who should be responsible, and this is an opportunity for further research.

### Clinical implications

A clinical trial may have several logistical challenges. GLP-1 receptor agonists have an expanding evidence base on their benefits to multiple co-morbidities and so a two-arm randomised controlled trial (RCT) comparing GLP-1 administration to a placebo group may not be ethical, especially if administration over a very long duration was required. This concern was also highlighted by our PPIE groups. The commercial availability of GLP-1 drugs with these publicly well-documented health benefits, may lead to significant contamination of a control arm, the cumulative effect of which may threaten the success of a clinical trial to elicit a true result. An RCT comparing a GLP-1 receptor agonist with another weight-loss intervention would require even greater sample sizes, if we presume any cancer protective effect is driven solely by weight-loss. Due to the rarity of cancer incidence, even a large relative risk reduction of 25%, in a UK/EU population, where baseline cancer risk is 7–8% over 10 years, would require over 7000 participants based on standard power calculation methodology. The likely significant contamination could drive this required sample size up higher, to logistically and financially infeasible levels.

Clearly, there are significant challenges in undertaking a trial to influence obesity-related cancer prevention clinical practice. However, the impact of a GLP-1-based chemoprevention strategy could be considerable. This is highlighted by a study of the OncoSim Canada database, estimating that over 70,000 cancers could be prevented over 25 years within the country using GLP-1 receptor agonists for the treatment of obesity on a population scale [28].

### Unanswered questions and future research

A thorough assessment of feasibility, alongside international collaboration, will help to pave the way for the best route to generating evidence to progress obesity-related cancer prevention in the future. This consensus is the first part of understanding the importance of this aspect of cancer research in the midst of a global obesity epidemic. Whilst this study has helped to select key aspects of such a trial, it will still be lengthy, complex and financially costly. There remain key uncertainties on the single best way to design a clinical trial and whether it is truly feasible to undertake in the real world. Further feasibility assessments including simulation modelling, accumulating observational evidence and pilot studies, may help to reach a definitive answer and provide the best chance of progressing the field of obesity-related cancer prevention. If a clinical trial is not feasible, an understanding of a theoretically ideal RCT might still be useful when applying the target trial framework [29] to the analysis of observational data.

## Supplementary information

Supplementary materials

## Data Availability

Full Delphi consensus results are available in Supplementary Table 3. Further anonymised data may be made available upon request.
