# Supplementary material for: Need for and design of a trial to test efficacy of weight loss interventions for cancer prevention: an international consensus using expert nominal group and Delphi methods
Source: Br J Cancer. 2026 Mar 2;134(8):1123–30. doi: 10.1038/s41416-026-03356-7 (PMC13036015; doi:10.1038/s41416-026-03356-7)
Supplement: Supplementary file 1 — Supplementary materials [file 41416_2026_3356_MOESM1_ESM.docx]

**Supplementary materials:**

**Need for and design of a trial to test efficacy of weight loss interventions for cancer prevention: an international consensus using expert nominal group and Delphi methods**

Matthew Harris,^1^ David P French,^2^ Ken Clare,^3^ Michelle Harvie,^1,4^ Duncan T Wilson,^5^ Julia Brown,^5^ David Jayne,^6^ Andrew G Renehan^1^

**Supplementary table 1: Participants in nominal group meeting 1**

| **Salutation** | **Name** | **Institution** |
| --- | --- | --- |
| Professor | Andrew Renehan | University of Manchester |
| Dr | Michelle Harvie | University of Manchester |
| Professor | David French | University of Manchester |
| Professor | David Jayne | University of Leeds |
| Professor | Julia Brown | University of Leeds |
| Dr | Duncan Wilson | University of Leeds |
| Professor | Jason Halford | University of Leeds |
| Professor | Amy Ahern | University of Cambridge |
| Professor | Annie Anderson | University of Dundee |
| Professor | Helen Croker | World Cancer Research Fund |
| Professor | Alex Miras | Ulster University |
| Professor | Richard Martin | University of Bristol |
| Professor | Karen Brown | University of Leicester |
| Professor | Marc Gunter | Imperial College London |
| Dr | Pietro Ferrari | IARC |
| Dr | Dimitris Papamargaritis | University of Leicester |
| Dr | Anisa Poljo | University Hospital Basel |
| Dr | Matthew Harris | University of Manchester |
| Mr | Ken Clare | Patient representative |

**Supplementary table 2 – Delphi consensus participants**

| **Salutation** | **Name** | **Institution** |
| --- | --- | --- |
| Prof | David French | University of Manchester |
| Prof | Annie Anderson | University of Dundee |
| Prof | Dimitris Papamargaritis | University of Leicester |
| Prof | Carel LeRoux | University College Dublin |
| Prof | Barbara Mcgowan | Kings College London |
| Dr | Helen Clarke | University of Manchester |
| Prof | Nils Halberg | University of Bergen |
| Prof | Kate McBride | Western Sydney University |
| Prof | Sarah Lewis | University of Bristol |
| Dr | Sixten Harborg | Aarhus University |
| Prof | Karen Brown | University of Leicester |
| Prof | Helen Croker | World Cancer Research Fund |
| Prof | Luca Busetto | University of Padova |
| Prof | Alex Miras | Universtiy of Ulster |
| Prof | Stephen Hurtsing | University of North Carolina |
| Prof | Helen Heneghan | University College Dublin |
| Prof | Chetan Parmar | University College London |
| Prof | Kath Williams | Western Sydney University |
| Prof | Martin Wiseman | World Cancer Research Fund |
| Prof | Marc Gunter | Imperial College |
| Prof | Jason Halford | University of Leeds |
| Dr | Maria Rohm | Helmholtz Munich |
| Dr | Matthew Harris | University of Manchester |
| Dr | Adisa Poljo | University of Basel |
| Dr | Sarah Kitson | University of Manchester |
| Dr | Mauricio Berrel Diaz | Helmholtz Munich |
| Prof | Emma Vincent | University of Bristol |
| Dr | Alicia Heath | Imperial College |
| Prof | Ralph Peterli | University of Basel |
| Prof | Marco Beuter | University of Zurich |
| Prof | Katharina Timper | University of Basel |
| Prof | Styliani Manziari | Lausanne University hospital |
| Prof | Pietro Ferrari | International Agency for Research on Cancer |
| Prof | Marco Von Strauss | University of Basel |
| Dr | Sam Orange | University of Newcastle |
| Prof | Michelle Harvie | University of Manchester |
| Prof | Patrick Folie | Kantonnspital St Gallen |
| Prof | Rebecca Beeken | University of Leeds |
| Dr | Laurence Dobbie | Kings College London |
| Prof | Tanja Stocks | Lund University |
| Prof | Fabio Garofalo | University hospital Basel |
| Prof | Signe Borgquist | Aarhus University |
| Prof | Piya Sengupta | Kings College London |
| Dr | Bethan Lloyd-Lewis | University of Bristol |
| Prof | Andres Acosta | Mayo clinic |
| Prof | Markus Gass | University hospital Basel |
| Prof | Pouya Iranmanesh | University of Geneva |
| Dr | Julia Muehlhaeusser | UH Lucerne |
| Prof | Stephan Herzig | Helmholtz Munich |
| Dr | Maria Daniela Hurtado | Mayo Clinic |
| Prof | Andrew Renehan | University of Manchester |
| Prof | Jack Cuzick | Queen Mary University |
| Prof | Richard Martin | University of Bristol |
| Prof | John Wilding | University of Liverpool |
| Prof | Julia Brown | University of Leeds |

**Supplementary table 3: Full Delphi consensus results**

| **Section 1:** **Need for a clinical weight loss to prevent cancer clinical trial to confirm that intentional weight loss can reduce cancer risk** | | | | | | | |
| --- | --- | --- | --- | --- | --- | --- | --- |
| Number | Statement | Round | Strongly agree | Agree | Neither agree nor disagree | Disagree | Strongly disagree |
| 1 | The current evidence for the causal link between weight loss intervention and cancer is strong enough to inform policy | 1 | 21.2% | 23.1% | 9.6% | 46.2% | 0% |
|  |  | 2 | 8.7% | 26.1% | 6.5% | 53.4% | 4.3% |
|  |  | 3 | 8.3% | 18.8% | 6.3% | 62.5% | 4.2% |
| 2 | Excess adiposity is causally associated with several major cancer types | 1 | 82.7% | 15.4% | 0% | 1.9% | 0% |
|  |  | 2 | Reached consensus | | | | |
|  |  | 3 |  |  |  |  |  |
| 3 | The causal pathway from excess adiposity to cancer development is likely to take at least a decade to manifest clinically for most cancer types | 1 | 21.2% | 50% | 23.1% | 5.8% | 0% |
|  |  | 2 | Reached consensus | | | | |
|  |  | 3 |  |  |  |  |  |
| 4 | A clinical trial is necessary to advance the current evidence for the causal effect for weight loss intervention and cancer risk reduction | 1 | 51.9% | 35.8% | 3.8% | 5.8% | 0% |
|  |  | 2 | Reached consensus | | | | |
|  |  | 3 |  |  |  |  |  |
| 5 | Putative pre-cancer biomarkers (e.g. blood samples, imaging) should be recorded throughout a trial investigating weight loss to prevent cancer | 1 | 63.5% | 26.9% | 9.6% | 0% | 0% |
|  |  | 2 | Reached consensus | | | | |
|  |  | 3 |  |  |  |  |  |
| 6 | A clinical trial investigating weight loss intervention to prevent cancer should aim to achieve a duration of follow up of at least 10 years | 1 | Question added in round 2 | | | | |
|  |  | 2 | 30.4% | 63% | 2.2% | 4.3% | 0% |
|  |  | 3 | Reached consensus | | | | |

| **Section 2: Population to include in a weight loss intervention to prevent cancer clinical trial** | | | | | | | |
| --- | --- | --- | --- | --- | --- | --- | --- |
| Number | Statement | Round | Strongly agree | Agree | Neither agree nor disagree | Disagree | Strongly disagree |
| 7a | A clinical trial for weight loss intervention to prevent cancer should assess effects on a single specific cancer type | 1 | 9.6% | 26.9% | 26.9% | 28.8% | 7.7% |
|  |  | 2 | Reworded for round 2 (question 8) | | | | |
|  |  | 3 |  |  |  |  |  |
| 7b | A clinical trial for weight loss intervention to prevent cancer should assess effects on a population with a high risk for a specific, individual, obesity-related cancer (e.g endometrial cancer in women with class 2 or 3 obesity), but examine incidence of any cancer within this population | 1 | Reworded into round 2 | | | | |
|  |  | 2 | 19.6% | 45.7% | 6.5% | 26.1% | 2.2% |
|  |  | 3 | 12.5% | 75% | 6.3% | 6.3% | 0% |
| 8 | A clinical trial should include a broad demographic representative of a general UK/EU population (considering ethnicity and socio-economic status) | 1 | 53.8% | 38.5% | 7.7% | 0% | 0% |
|  |  | 2 | Reached consensus | | | | |
|  |  | 3 |  |  |  |  |  |
| 9 | A clinical trial should include participants in a single country or geographical area | 1 | 0% | 13.5% | 25% | 55.8% | 3% |
|  |  | 2 | 0% | 8.7% | 10.9% | 78.3% | 2.2% |
|  |  | 3 | Reached consensus | | | | |
| 10a | A clinical trial should consider including only participants with a high baseline risk of cancer (e.g Lynch, BRCA, or snp scores) to increase baseline incidence and power of the study | 1 | 11.5% | 15.9% | 23.1% | 42.3% | 7.7% |
|  |  | 2 | 4.3% | 15.2% | 10.9% | 63% | 6.5% |
|  |  | 3 | Reworded for round 3 (question 12) | | | | |
| 10b | A clinical trial should include **only** participants with a high baseline risk of cancer [e.g Lynch, BRCA, or snp scores] to increase baseline cancer incidence and power of the study | 1 | Reworded into round 3 | | | | |
|  |  | 2 |  |  |  |  |  |
|  |  | 3 | 2.1% | 18.8% | 0% | 70.8% | 8.3% |

| Question  11 | Participants with established obesity-related comorbidity [defined as T2DM, cardiovascular disease] should be: | Round | Included alongside non-comorbid | Excluded with non-comorbid included | Included with non-comorbid excluded |
| --- | --- | --- | --- | --- | --- |
|  |  | 1 | 90.4% | 7.7% | 1.9% |
|  |  | 2 | Reached consensus | | |
|  |  | 3 |  |  |  |

| Question  12 | A clinical trial for weight loss intervention and cancer risk reduction should take place in this setting: | Round | Primary care | Secondary care | Specialist weight management service | Any setting |
| --- | --- | --- | --- | --- | --- | --- |
|  |  | 1 | 15.4% | 5.8% | 19.2% | 57.7% |
|  |  | 2 | 17.4% | 4.3% | 15.2% | 60.9% |
|  |  | 3 | 6.3% | 12.5% | 2.1% | 91.7%* |

*answer re-worded to Any setting (depends on intervention and population)

| **Section 3: Intervention for a weight loss intervention to prevent cancer clinical trial** | | | | | | | | | | | |
| --- | --- | --- | --- | --- | --- | --- | --- | --- | --- | --- | --- |
| Number | Statement | Round | Strongly agree | | Agree | | Neither agree nor disagree | | Disagree | | Strongly disagree |
| 13a | A weight loss to prevent cancer trial should investigate a single specific intervention approach (e.g Semaglutide SC, or behavioural programme with diet and/or physical activity modification) | 1 | 11.5% | | 19.2% | | 9.6% | | 48.1% | | 11.5% |
|  |  | 2 | Reworded for round 2 | | | | | | | | |
|  |  | 3 |  |  |  |  |  |  |  |  |  |
| 13b | A clinical trial should investigate the effect of single specific interventions on cancer risk (E.g semaglutide SC, semaglutide + behavioural intervention, tirzepatide etc, rather than 'weight-loss intervention' in general). This may be a trial design comparing multiple interventions such as a platform trial. | 1 | Reworded for round 2 | | | | | | | | |
|  |  | 2 | 13% | 41.3% | | 4.3% | | 39.1% | | 2.2% | |
|  |  | 3 | 14.6% | 66.7% | | 6.3% | | 12.5% | | 0% | |
| 14 | A platform trial* including multiple weight loss intervention types should be considered as a solution to allow the inclusion of multiple interventions | 1 | 32.7% | 51.9% | | 9.6% | | 5.8% | | 0% | |
|  |  | 2 | Reached consensus | | | | | | | | |
|  |  | 3 |  |  |  |  |  |  |  |  |  |
| 15 | If GLP-1/dual receptor agonists are used as a clinical trial intervention, participants should have drug administration for the entire duration of follow up (5-15 years) | 1 | 17.3% | 34.6% | | 28.8% | | 19.2% | | 0% | |
|  |  | 2 | 19.6% | 39.1% | | 23.9% | | 17.4% | | 0% | |
|  |  | 3 | 12.5% | 50% | | 31.3% | | 6.3% | | 0% | |
| 16 | If GLP-1/dual receptor agonists are considered as the intervention, a clinical trial should give participants GLP-1/dual receptor agonist administration for up to 2 years and then alternative ‘maintenance intervention’ | 1 | 3.8% | 42.3% | | 25% | | 21.2% | | 7.7% | |
|  |  | 2 | 2.2% | 50% | | 19.6% | | 23.9% | | 4.3% | |
|  |  | 3 | 4.2% | 64.6% | | 6.3% | | 22.9% | | 2.1% | |
| 17 | In a clinical trial investigating the impact of bariatric surgery on cancer risk, a control group receiving no weight loss intervention would be unethical | New question in round 2 | | | | | | | | | |
|  |  | 2 | 15.2% | 52.2% | | 15.2% | | 13% | | 4.3% | |
|  |  | 3 | 16.7% | 58.3% | | 8.3% | | 14.6% | | 2.1% | |
| 18 | In a clinical trial investigating the impact of pharmacotherapy on cancer risk, a control group receiving no weight loss intervention and placebo would be unethical | New question in round 2 | | | | | | | | | |
|  |  | 2 | 13% | 37% | | 17.4% | | 28.3% | | 4.3% | |
|  |  | 3 | 6.3% | 50% | | 14.3% | | 27.1% | | 2.1% | |
| 19a | If GLP-1/dual receptor agonists are considered as the intervention, a clinical trial should allow participants to go on and off the drug during the duration of the trial | New question in round 2 | | | | | | | | | |
|  |  | 2 | 2.2% | 34.8% | | 19.6% | | 30.4% | | 13% | |
|  |  | Question reworded for round 3 | | | | | | | | | |
| 19b | If GLP-1/dual receptor agonists are considered as the intervention, participants should have the option of being on the medication for the duration of the trial, but with the option to step off the drug once weight loss has been achieved and stabilised, reflecting real-world practices | Question reworded in round 3 | | | | | | | | | |
|  |  | 3 | 8.3% | 54.2% | | 10.4% | | 22.9% | | 4.2% | |

| Question 20 | Rank each category of weight loss intervention based on appropriateness for use in a weight loss intervention to prevent cancer clinical trial (with rank 1 being most preferred) |
| --- | --- |
| 1 | 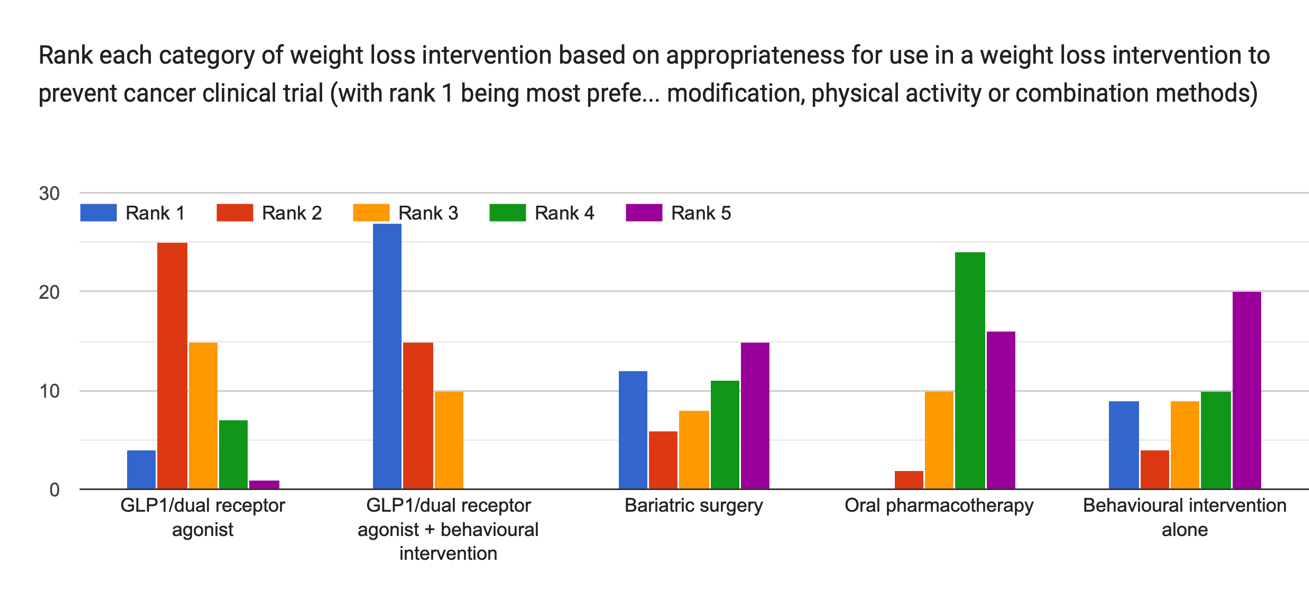 |
| 2 | 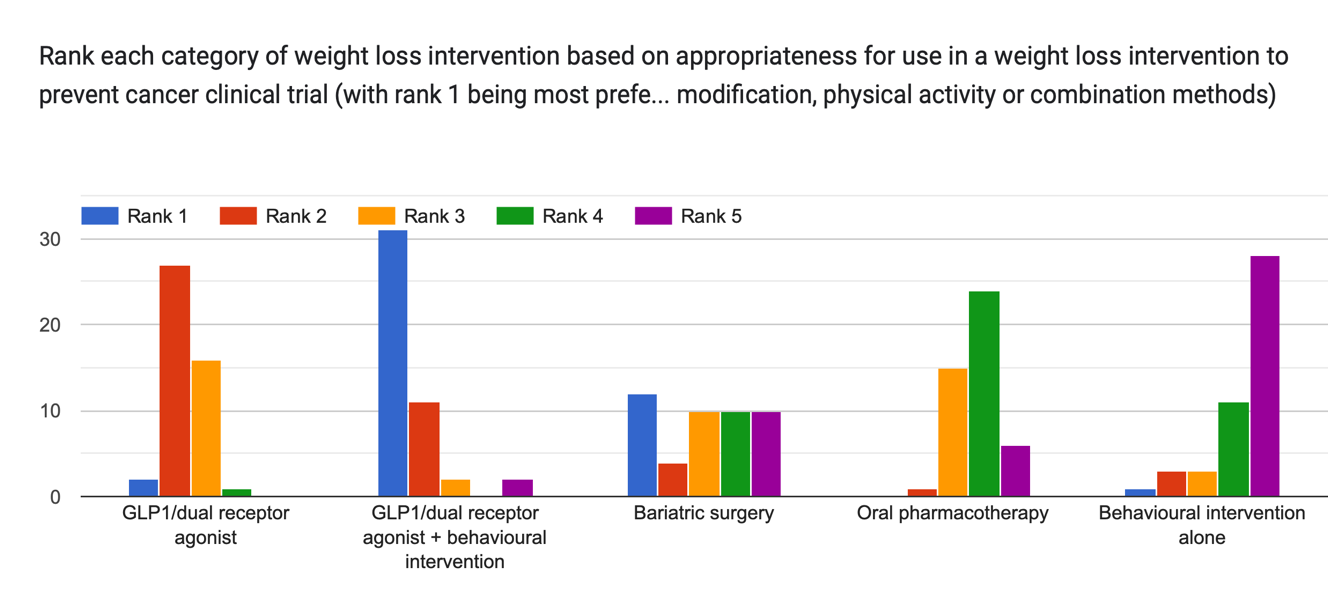 |
| 3 | 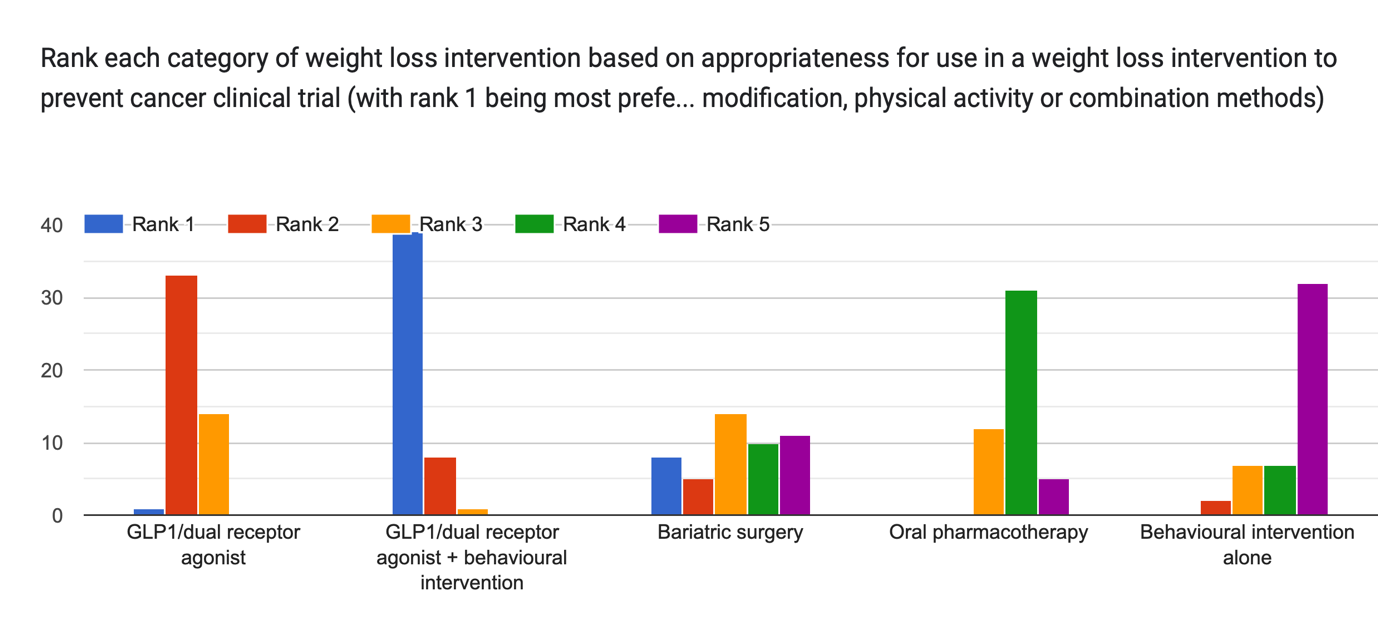 |

| Number  21 | The target total sustained body weight loss for a clinical trial of weight loss intervention for cancer prevention should be: | Round | <2% | 2-5% | 5-10% | 10-15% | 15-20% | >20% | No weight target |
| --- | --- | --- | --- | --- | --- | --- | --- | --- | --- |
|  |  | 1 | 0% | 0% | 28.8% | 42.3% | 19.2% | 9.6% | Added R2 |
|  |  | 2 | N/a | N/a | 10.9% | 63% | 21.7% | N/a | 4.3% |
|  |  | 3 | N/a | N/a | 8.3% | 66.7% | 20.8% | N/a | 4.2% |

| **Section 4: Control group to include in a weight loss intervention to prevent cancer clinical trial** | | | | | | | | | | | |
| --- | --- | --- | --- | --- | --- | --- | --- | --- | --- | --- | --- |
| Number | Statement | Round | Strongly agree | Agree | | Neither agree nor disagree | | | Disagree | | Strongly disagree |
| 22 | In a weight loss intervention to prevent cancer trial, a control group with no weight loss intervention in this context is unethical | 1 | 17.3% | 30.8% | | 17.3% | | | 28.8% | | 5.8% |
|  |  | 2 | 10.9 | 43.5% | | 10.9% | | | 32.6% | | 2.2% |
|  |  | 3 | Question removed following feedback from participants. Changed to questions 16 and 17. | | | | | | | | |
| 23 | A control group for a weight-loss intervention to prevent cancer trial with a GLP-1/dual receptor agonist as the intervention should be: | 1 | Consensus could not be reached. Following feedback from participants, questions 22 to 25 were removed and replaced with question 26.  (Responses are available in figures X to X) | | | | | | | | |
|  |  | 2 |  |  |  |  |  |  |  |  |  |
|  |  | 3 |  |  |  |  |  |  |  |  |  |
| 24 | A control group for a weight-loss intervention to prevent cancer trial, with a GLP-1/dual receptor agonist PLUS behavioural intervention as the intervention should be: | 1 | Consensus could not be reached. Following feedback from participants, questions 22 to 25 were removed and replaced with question 26. (Responses are available in figures X to X) | | | | | | | | |
|  |  | 2 |  |  |  |  |  |  |  |  |  |
|  |  | 3 |  |  |  |  |  |  |  |  |  |
| 25 | A control group for a weight-loss intervention to prevent cancer trial with bariatric surgery as the intervention should be: | 1 | Consensus could not be reached. Following feedback from participants, questions 22 to 25 were removed and replaced with question 26. (Responses are available in figures X to X) | | | | | | | | |
|  |  | 2 |  |  |  |  |  |  |  |  |  |
|  |  | 3 |  |  |  |  |  |  |  |  |  |
| 26 | A control group in a weight-loss intervention to prevent cancer trial with behavioural change intervention as the intervention should be: | 1 | Consensus could not be reached. Following feedback from participants, questions 22 to 25 were removed and replaced with question 26. (Responses are available in figures X to X) | | | | | | | | |
|  |  | 2 |  |  |  |  |  |  |  |  |  |
|  |  | 3 |  |  |  |  |  |  |  |  |  |
| 27 | Selection of a control group depends on the intervention, population and setting selected for a clinical trial | 1 | New question added in round 3 | | | | | | | | |
|  |  | 2 |  |  |  |  |  |  |  |  |  |
|  |  | 3 | 25% | | 75% | | 0% | 0% | | 0% | |

| **Section 5: Outcome for a weight loss intervention to prevent cancer clinical trial** | | | | | | | |
| --- | --- | --- | --- | --- | --- | --- | --- |
| Number | Statement | Round | Strongly agree | Agree | Neither agree nor disagree | Disagree | Strongly disagree |
| 28 | Multiple primary end points should be considered e.g cancer mortality, overall mortality, incidence of type 2 diabetes | 1 | 34.6% | 40.4% | 7.7% | 15.4% | 2.2% |
|  |  | 2 | Reached consensus | | | | |
|  |  | 3 |  |  |  |  |  |
| 29 | Surrogate/precursor endpoints for cancer incidence should be considered | 1 | 87.2% | 15.4% | 0% | 1.9% | 0% |
|  |  | 2 | Reached consensus | | | | |
|  |  | 3 |  |  |  |  |  |

| Question  30 | The primary outcome for a weight loss to prevent cancer trial should be: | Round | Absolute incidence of any cancer | Absolute incidence of obesity-related cancer | Incidence of a specific cancer | Time to cancer |
| --- | --- | --- | --- | --- | --- | --- |
|  |  | 1 | 36.5% | 44.2% | 13.5% | 3.8% |
|  |  | 2 | 41.3% | 52.2% | 4.3% | 2.2% |
|  |  | 3 | 33.3% | 58.3% | 8.3% | 0% |

| Question 31 (added in round 2) | Please select from below which primary endpoints should be selected for a weight loss intervention to prevent cancer clinical trial, if multiple primary end points were planned | Round 2 | Round 3 |
| --- | --- | --- | --- |
|  | Incidence of any cancer | 65.2% | 75% |
|  | Incidence of obesity-related cancer | 84.8% | 79.2% |
|  | Overall mortality | 52.2% | 27.1% |
|  | Cancer mortality | 69.9% | 60.4% |
|  | Cardiovascular disease incidence | 32.6% | 10.4% |
|  | Obesity-related disease incidence | 30.4% | 18.8% |
|  | Pre-cancer incidence (E.g Barretts) | 37% | 14.6% |

| Question 32 (added in round 3) | Please select from below which secondary endpoints should be selected for a weight loss intervention to prevent cancer clinical trial | Round 3 |
| --- | --- | --- |
|  | Cancer pre-cursor incidence | 66.7% |
|  | Weight loss | 77.1% |
|  | Overall mortality | 64.6% |
|  | Cancer mortality | 41.7% |
|  | Cardiovascular disease incidence | 60.4% |
|  | Obesity-related disease incidence | 64.6% |
|  | Insulin resistance of other relevant marker | 50.7% |

| **Section 6: Research priority areas** | | | | | | | |
| --- | --- | --- | --- | --- | --- | --- | --- |
| Number | Statement | Round | Strongly agree | Agree | Neither agree nor disagree | Disagree | Strongly disagree |
| In round 1, participants were asked: ‘In nominal group meeting 1, there was agreement that there is a lack of understanding of causal pathways between weight loss intervention and cancer risk reduction. In your opinion, what are the highest priority areas of research in understanding the relationship between weight loss intervention and cancer prevention?’  Free text answers were analysed to produce 6 further statements in round 2. | | | | | | | |
| 33 | A research priority in the area of weight loss intervention and cancer risk is: research to understand the biological causal pathways between specific weight loss interventions and the effect on cancer development | New question in round 2 | | | | | |
|  |  | 2 | 45.7% | 45.7% | 6.5% | 2.2% | 0% |
|  |  | 3 | Reached consensus | | | | |
| 34 | A research priority in the area of weight loss intervention and cancer risk is: research at a molecular level to understand the biological causal pathways between intentional weight loss and the effect on cancer development | New question in round 2 | | | | | |
|  |  | 2 | 41.3% | 50% | 2.2% | 6.5% | 0% |
|  |  | 3 | Reached consensus | | | | |
| 35 | A research priority in the area of weight loss intervention and cancer risk is: epidemiological studies exploring intentional weight loss and cancer risk | New question in round 2 | | | | | |
|  |  | 2 | 21.7% | 50% | 8.7% | 19.6% | 0% |
|  |  | 3 | Reached consensus | | | | |
| 36 | A research priority in the area of weight loss intervention and cancer risk is: the identification of cancer precursor markers affected in weight loss | New question in round 2 | | | | | |
|  |  | 2 | 32.6% | 58.7% | 4.3% | 4.3% |  |
|  |  | 3 | Reached consensus | | | | |
| 37 | A research priority in the area of weight loss intervention and cancer risk is: research exploring the microbiome and its relationship with weight loss and cancer risk | New question in round 2 | | | | | |
|  |  | 2 | 10.9% | 30.4% | 34.8% | 21.7% | 2.2% |
|  |  | 3 | 4.2% | 39.6% | 29.2% | 22.9% | 4.2% |
| 38 | A research priority in the area of weight loss intervention and cancer risk is: research exploring the effect of intentional weight loss on hormonal and metabolic pathways, systemic inflammation and these effects on cancer development | New question in round 2 | | | | | |
|  |  | 2 | 37% | 52.2% | 8.7% | 2.2% | 0% |
|  |  | 3 | Reached consensus | | | | |

**Supplementary table 4 – Final nominal group participants**

| **Name** | **Affiliation** |
| --- | --- |
| Matt Harris | University of Manchester |
| Andrew Renehan | University of Manchester |
| Michelle Harvie | University of Manchester |
| David French | University of Manchester |
| John Wilding | University of Liverpool |
| Stephen Hursting | University of North Carolina |
| Helen Henghan | University College Dublin |
| Rebecca | University of Leeds |
| Helen Clarke | University of Manchester |
| Sam Orange | University fo Newcastle |
| Annie Anderson | University of Dundee |
| Sixten Harborg | Aarhus University |
| Emma Vincent | University of Bristol |
| Helen Croker | World Cancer Research Fund |
| Alicia Heath | Imperial College |
| Martin Wiseman | World Cancer Research Fund |
| Tanja Stocks | Lund Univesity |
| Luca Busetto | University of Padoiva |
| Richard Martin | University of Bristol |
| Jason Halford | University of Leeds |
| Kate McBride | Western Sydney Univerisity |
| Karen Brown | University of Leicester |
| Mauricio Berriel Diaz | Helmholz Munich |
| Duncan Wilson | University of Leeds |
| Dimitris Papamargaritis | University of Leicester |
| Bethan Lloyd-Lewis | University of Bristol |
| Piya Sen Gupta | Kings College London |
| Daniela Hurtado | Mayo Clinic |
| Maria Rohm | Helmholz Munich |
| Katharina Timper | University of Basel |
